# Clinical characteristics and treatment outcomes in Camurati–Engelmann disease: A case series

**DOI:** 10.1097/MD.0000000000010309

**Published:** 2018-04-06

**Authors:** Yoon-Myung Kim, Eungu Kang, Jin-Ho Choi, Gu-Hwan Kim, Han-Wook Yoo, Beom Hee Lee

**Affiliations:** a Department of Pediatrics; b Medical Genetics Center, Asan Medical Center Children's Hospital, University of Ulsan College of Medicine, Seoul, Republic of Korea.

**Keywords:** Camurati–Engelmann disease, progressive diaphyseal dysplasia, transforming growth factor beta 1

## Abstract

**Background:**

Camurati–Engelmann disease is an extremely rare disease characterized by hyperostosis of multiple long bones. This condition is caused by heterozygous mutations in the *TGFB1* gene.

**Methods:**

We describe the clinical and genetic characteristics of 4 Korean patients with this rare disease diagnosed at Asan Medical Center in Korea between June 2012 and May 2016, to increase awareness about this condition among general physicians and orthopedists. The presenting features, biochemical findings, radiographic and nuclear imaging findings, molecular analysis, and treatment outcomes of 4 patients were reviewed retrospectively.

**Results:**

Two patients had sporadic disease, whereas the other 2 were familial cases. The average age at symptom onset was 8.8 ± 5.5 (4–14) years. Symptoms included waddling gait or leg pain. Bone pain and easy fatigability were documented in all patients. Skeletal deformities such as osteoporosis, genu valgum, and severe scoliosis were observed. Visual and otologic manifestations presenting as exophthalmos, retinal detachment, and vestibulopathy were found in 3 patients. Skeletal survey showed diaphyseal expansion with diffuse cortical thickening of long bones in all patients. Bone scintigraphy images showed increased uptake of radioactive material in the calvarium and diaphysis of long bones. The mean erythrocyte sedimentation rate was 46.5 ± 22.2 (20–72) mm/h. Sequence analysis of *TGFB1* revealed the previously reported mutations p.Arg218His, p.Arg218Cys, and p.Glu169Lys. Corticosteroid was effective in relieving pain, and losartan was used as maintenance therapy.

**Conclusions:**

Our experience suggests that this rare condition can be suspected in patients with characteristic symptoms and skeletal findings. Considering the presence of effective medical treatment, efforts are needed to identify more cases.

## 1 Introduction

Camurati–Engelmann disease (CED; OMIM: 131300), or progressive diaphyseal dysplasia, is a rare congenital disease that is characterized by hyperostosis of multiple long bones. Patients with CED experience bone pain in their extremities, with skeletal muscle weakness from childhood.^[1]^ They usually start to walk later than unaffected children, and waddling gait is noted in some patients. The characteristic radiographic finding is hyperostosis of the diaphyses of long bones, which may progress into the metaphyses and, rarely, the epiphyses.^[1–3]^ Diffuse and severe thickening of the bony calvarium and skull base is also a common finding, which may bring visual and otologic manifestations in some patients with basal skull sclerosis.^[4–7]^

The pathogenesis of this disease is associated with the abnormalities in intramembranous bone formation. Mutations in the coding region for the latency-associated peptide (LAP) in the transforming growth factor beta 1 (*TGFB1*) gene cause CED through gain-of-function effects.^[8,9]^ Normally, TGF-β1 is separated from LAP and released as an activated form at the sites of bone resorption to stimulate bone formation.^[9]^ However, in CED, mutations in the LAP cause premature dissociation of TGF-β1 causing distortion of the resorption-induced TGF-β1 gradients. The inadequate activation of TGF-β1 on unnecessary sites leads to poor-quality bone formation, unfilled resorbed areas, and haphazard sclerotic areas.^[9]^ Understanding the pathophysiology of the disease provided important insights into its treatment strategies; currently, corticosteroids and angiotensin receptor II blockers are recommended to improve the clinical outcome of patients with CED.^[10,11]^

Despite the distinctive radiological features and pan-ethnic characteristics, only >300 patients with CED have been reported,^[11]^ and CED is considered a rare disease with an unknown prevalence. Considering the existence of effective treatment modalities, early diagnosis is important to improve the clinical outcome of affected patients, as well as their quality of life.

Herein, the clinical features and radiological characteristics of CED are described in 4 Korean patients. The clinical outcomes of these patients were also assessed to evaluate the efficacy and adequacy of the currently recommended treatment methods. Our study aids in understanding the clinical and genetic characteristics of this rare genetic condition and the considerations in its management.

## 2 Materials and methods

### 2.1 Subjects

A total of 2 unrelated patients with sporadic CED and 2 patients with familial CED (3 men, 1 woman) diagnosed at Asan Medical Center in Korea between June 2012 and May 2016 were included in the current study. The diagnosis of CED was based on clinical and radiographic findings, and molecular analysis. This study was approved by the institutional review board of Asan Medical Center, Korea, and informed consent was obtained from all patients or their parents.

### 2.2 Molecular analysis

Genomic DNA was extracted from peripheral blood leukocytes by using a Puregene DNA isolation kit (Gentra, Minneapolis, MN). Direct sequencing of the *TGFB1* gene was performed with genomic DNA from peripheral blood leukocytes. All coding exons and exon-intron boundaries of the genes were individually amplified through polymerase chain reaction (PCR) by using primers (Table 1) designed from the flanking regions of each gene. Amplified PCR products were directly sequenced by using the BigDye Terminator v.3.1 Cycle Sequencing Kit and ABI3130x1 Genetic Analyzer (Applied Biosystems, Foster City, CA).

**Table 1:**
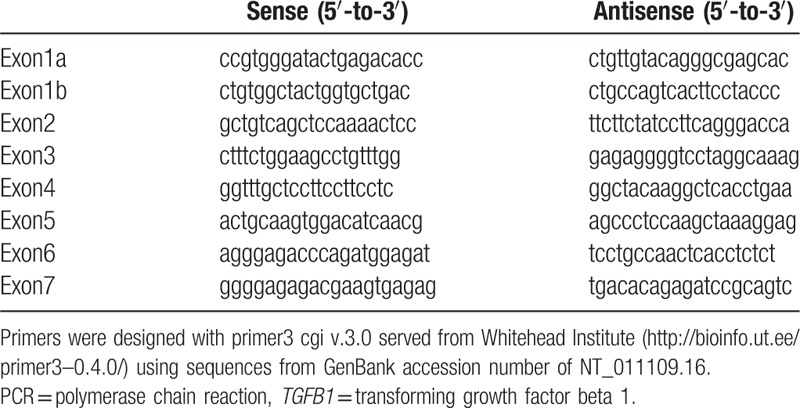
Sequences of primers using PCR reaction for *TGFB1* gene.

## 3 Results

### 3.1 Clinical features

**Figure 1:**
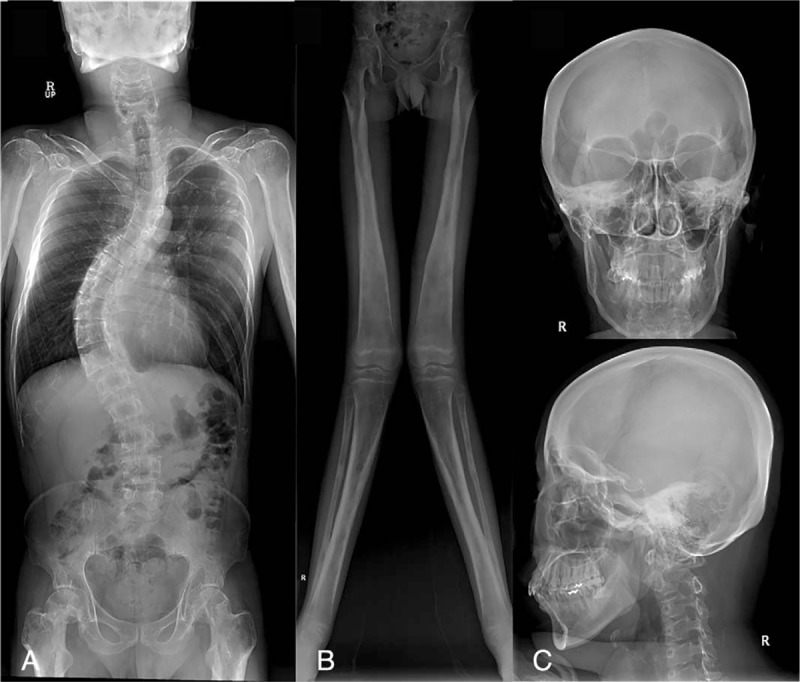
(A) Spine radiograph of patient 2. Note the severe thoracolumbar spine scoliosis at the T3–L3 level. (B) Lower-extremity radiographs of patient 1. Bilateral symmetrical hyperostosis and endostosis of the diaphysis were noted in both femurs, tibias, and fibulas. (C) Skull radiographs of patient 3 showing bilateral sclerotic bony enlargement in the skull base and calvarium.

Among the 3 men and 1 woman, patients 1 and 2 were sporadic cases and patients 3 and 4 were familial cases (Table 2). The average age at symptom onset was 8.8 ± 5.5 years (range, 4–14 years). The body mass index standard deviation score (BMI-SDS) was −2.4 ± 2.5 SDS at presentation and −1.8 ± 2.2 SDS at 1-year follow-up. Patients 1 and 4 presented with waddling gait at age 4 years, and patients 2 and 3 presented with bone pain in the lower extremities at age 13 and 14 years, respectively. In addition, all patients had muscle weakness and bone pain of the extremities with easy fatigability. Patient 1 received closed wedge corrective osteotomy at age 18 years owing to progressive genu valgum accompanying gait disability and knee pain. Patient 2 received surgical correction for severe thoracolumbar spine scoliosis (T3–L3 level) at age 35 years (Fig. 1A). Patients 3 and 4 had no specific skeletal deformities requiring surgical correction.

**Table 2:**
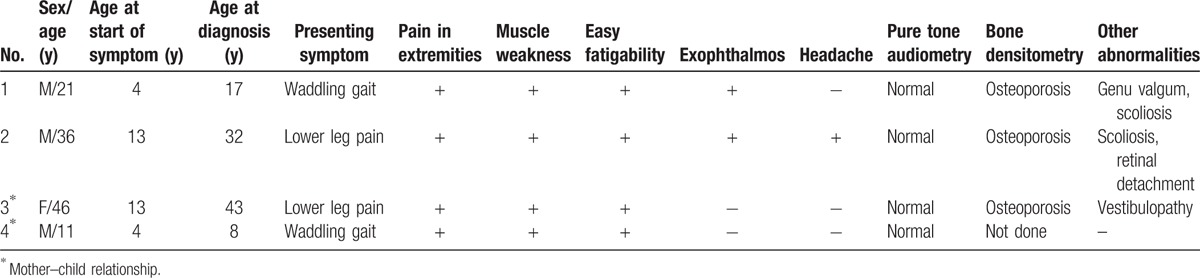
Clinical characteristics of patients.

Visual and otologic manifestations were observed in some patients. Patients 1 and 2 showed exophthalmos. In addition, patient 2 had blurred vision at age 29 years, and retinal detachment was found in the right eye. Patient 3 had dizziness occurring several times a day, and was found to have recurrent vestibulopathy on otologic examination. The results of pure tone audiometry were normal in all patients.

### 3.2 Imaging findings

**Figure 2:**
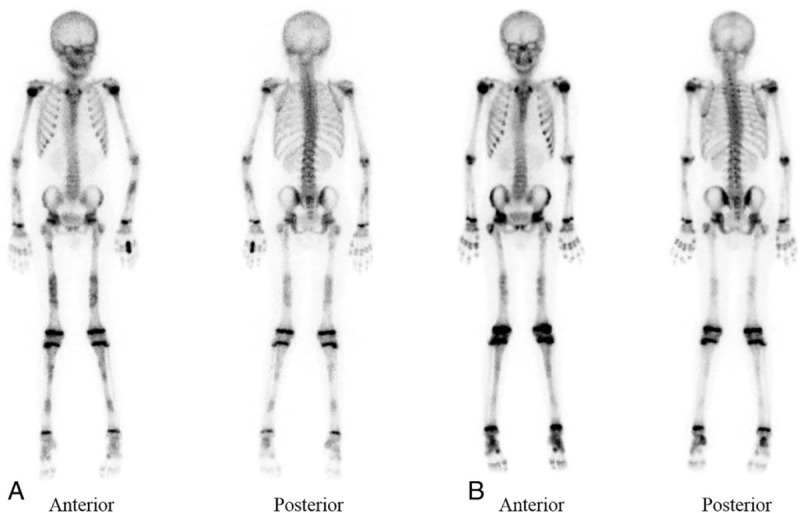
Bone scintigraphy images of patient 4. (A) Symmetric and diffuse inhomogeneously increased uptake in the long bones of both extremities were observed, which were consistent with the radiographic findings. (B) Decreased intensities of uptake of long bones diaphysis were observed after 1 year of treatment with deflazacort and losartan.

In all patients, bilateral symmetrical hyperostosis and endostosis of the diaphysis were noted in long bones such as the humeri, radii, ulnae, femurs, tibias, and fibulas (Fig. 1B). The long bones were elongated, and the medullary canal was narrowed. Skull radiographs demonstrated bilateral sclerotic bony enlargement in the skull base and calvarium, whereas they were normal in 1 patient (patient 4) at age 12 years (Fig. 1C). Bone scintigraphy revealed symmetric and diffuse inhomogeneously increased uptake in the long bones of both extremities and the skull, consistent with the radiographic findings (Fig. 2). Head computed tomography (CT) was taken in 2 patients and revealed narrow cranial nerve foramina, including the optic nerve canal, orbital fissure, and internal auditory canal. As osteoporosis has been reported in some patients,^[12,13]^ bone densitometry of the spine and femur was performed in 3 patients, which showed significantly reduced values consistent with osteoporosis.

### 3.3 Biochemical findings and molecular analysis

The biochemical findings at initial diagnosis are summarized in Table 3. The serum parathyroid hormone (PTH) and calcium levels were normal in all 4 patients (reference range, 10–65 pg/mL; calcium, 8.6–10.2 mg/dL). Alkaline phosphatase (ALP) was in the reference range except in patient 1 (330 IU/L), which was measured after surgical screw fixation of the distal tibia owing to genu valgum (reference range, male and female patients >15 years, 40–120 U/L). Sequence analysis of *TGFB1* identified p.Arg218His, p.Arg218Cys, and p.Glu169Lys mutations, all of which were previously reported as pathogenic.^[14,15]^

**Table 3:**
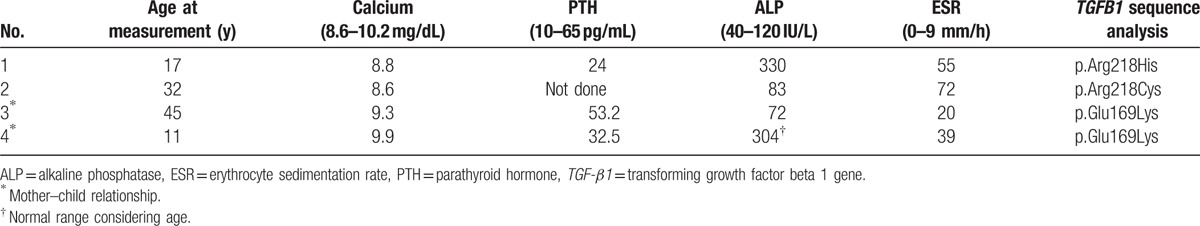
Biochemical and molecular findings at presentation.

### 3.4 Treatment outcomes

**Figure 3:**
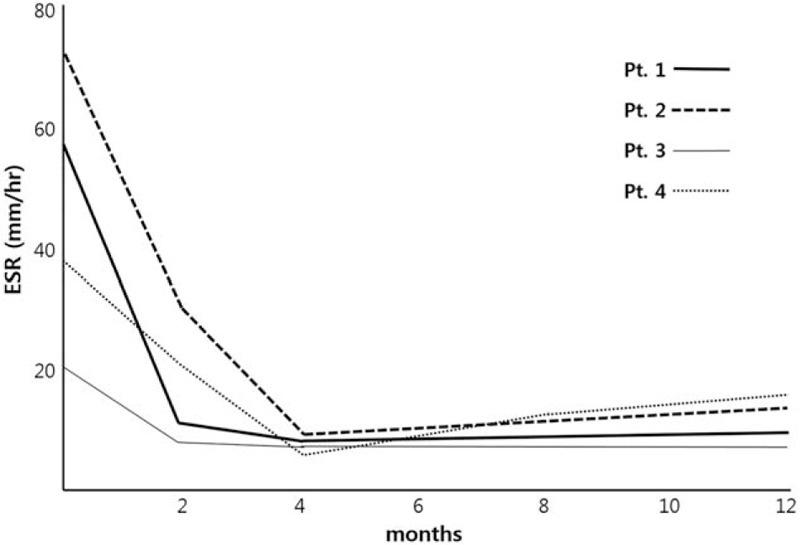
Trend of ESR after treatment. ESR = erythrocyte sedimentation rate.

Corticosteroid (deflazacort 1–1.2 mg kg^−1^ day^−1^) was used as initial therapy to relieve pain in all 4 patients. Age at the start of treatment was 26.5 ± 15.1 years (range, 11–45 years). In all patients, effective alleviation of acute pain was achieved after 1 to 3 months of therapy. Losartan (0.8–1.2 mg kg^−1^ day^−1^) was administrated subsequently as maintenance therapy in combination with corticosteroid. The dose of corticosteroid was decreased gradually when pain was controlled well. After 2 years of therapy, patient 1 was able to tolerate bone pain with losartan monotherapy, whereas patients 2, 3, and 4 experienced aggravation of bone pain or headache with the discontinuation of corticosteroid. Patients 2 and 3 complained of transient hypotension and dizziness with losartan, which were relieved with dose reduction of losartan. The mean erythrocyte sedimentation rate (ESR) of the 4 patients was 46.5 ± 22.2 mm/h (reference range, 0–9 mm/h) before the initiation of corticosteroid administration, but decreased to 18.8 ± 10.4 and 9.3 ± 1.0 mm/h at 2 and 4 months of treatment, respectively (Fig. 3). BMI-SDS was increased from −3.4 SDS to −1.5 SDS at 1-year follow-up in patient 2, whereas no prominent increments were observed in other patients. In patient 1, bone densitometry revealed improvement of osteoporosis after 4 years of treatment. Bone scintigraphy findings of patient 4 showed improvement after a year of treatment (Fig. 2). However, hyperostosis of diaphysis remained unchanged in all patients.

## 4 Discussion

The current study described the clinical features of 4 patients with CED, including their presenting signs, characteristic radiological features, and clinical courses with medical therapy. The diagnosis of CED was confirmed through genetic testing for *TGFB1*.

One of the most striking radiological features of CED is sclerosing bone dysplasia. However, sclerosing bone dysplasia can be caused by a variety of disorders showing defects in the bone ossification pathway.^[16]^ Sclerosing bone dysplasia can be categorized according to the presence of heredity and the site of abnormal bone formation. Hereditary sclerosing bone dysplasias include osteopetrosis, pyknodysostosis osteopoikilosis, osteopathia striata, progressive diaphyseal dysplasia (CED), hereditary multiple diaphyseal sclerosis (Ribbing disease), hyperostosis corticalis generalisata, and endosteal hyperostosis.^[16]^ The site of affected bones also depends on whether a defect affects the endochondral or intramembranous bone formation. CED is inherited in an autosomal dominant pattern and affects intramembranous bone formation.^[8,9,16]^ Therefore, the affected patients exhibit malformations of the skull and diaphysis of the long bones, as observed in our patients. The metaphyses and epiphyses, which are formed by endochondral bone formation, are generally spared. Increased uptake lesions appearing on bone scintigraphy findings were consistent with sclerosing dysplasia lesions on radiographs. Performing bone scintigraphy may be helpful for a patient presenting with extremity pain and suspected of having CED.^[17,18]^ Bone scintigraphy is a valuable diagnostic tool because increased tracer uptake can be perceived even before sclerosis becomes radiologically visible. There are several disorders that overlap clinical features of CED such as long bone sclerosis or cranial hyperostosis. These disorders include craniodiaphyseal dysplasia, Kenny–Caffey syndrome type 2, Juvenile Paget disease, Ghosal hemtodiaphyseal dysplasia, and endosteal hyperostosis.^[19]^ Distinctive features or certain laboratory findings identified in other long bone sclerosis disorders would be helpful for the differential diagnosis.^[19]^

In previous reports, the average age at symptom onset ranged from 9 to 13 years, with lower-extremity pain, gait unsteadiness, or ataxia, similar to our cases (8.8 ± 5.5 years).^[1,6]^ Headache and ophthalmopathy appear as early as in the second to third decade.^[6]^ Audiovestibular and facial nerve involvements manifest later in the fourth to fifth decade.^[6]^ The age at onset of visual and otological symptoms was the same as in previous reports. As presenting signs, extremity pain is the most common manifestation, followed by waddling gait, easy fatigability, and muscle weakness.^[3,6]^ All patients in our study also showed extremity pain, muscle weakness, and easy fatigability. Because CED is characterized by progressive sclerotic bony changes, gait abnormality is often observed in patients in their first decade of life.^[6]^ Other signs of musculoskeletal involvement include lumbar kyphosis, lordosis, scoliosis, coxa valga, genu valgum, and pes planus. The incidence of hearing impairment in CED is estimated to be up to 18%,^[20]^ although none of our patients showed abnormal PTA results; however, annual follow-up is needed considering previous reports.^[6]^ Visual manifestations of CED are considered late sequelae of the disease process.^[21]^ The visual symptoms are presumed to occur owing to compressive optic neuropathy or secondary to papilledema.^[4]^ Brain CT in the patient with retinal detachment in our report showed headache, exophthalmos, and narrowed cranial foramina. Retinal detachment in this patient is considered one of the late sequelae of CED. On the other hand, these progressive clinical manifestations vary among patients owing to variable expressivity and penetrance.

In CED, the serum calcium, phosphorus, ALP, and PTH levels are normal, as in our study. The only characteristic biochemical profile is elevated ESR, which was observed in all 4 patients. Some reports also described elevation of ALP.^[1]^ The identification of CED has been problematic despite its characteristic radiological and biochemical findings, owing to its rarity and diverse clinical spectrum among patients. Although the disease is inherited in an autosomal dominant manner, it occurs sporadically in about 30% of patients with CED.^[6]^ Molecular genetic testing is necessary in suspected cases to confirm the diagnosis. Most mutations are missense, gain-of-function mutations in exon 4 leading to single amino acid substitutions in the encoded protein.^[3,14]^ p.Arg218Cys has been the most prevalent mutation, followed by p.Cys225Arg and p.Arg218His. The clinical findings of patients carrying these 3 mutations did not seem to differ significantly.^[14]^ p.Arg218Cys and p.Arg218His mutations were also identified in our 2 sporadic cases. Less frequently, mutations in exon 1 or 2 have also been reported.^[3,14]^ A familial case of p.Glu169Lys mutation in exon 2, which was also revealed in our familial case, was reported in a Chinese family.^[15]^ Thus far, there is no known genotype–phenotype correlation.

Concerning pharmacological treatment, corticosteroids decrease bone density by suppressing osteoblast proliferation, differentiation, and bone matrix synthesis.^[22]^ On the other hand, they enhance the proliferation and differentiation of osteoclast precursors.^[23]^ In patients with CED, glucocorticoids work as an opposing agent of bone formation. There are several reports demonstrating the successful treatment of CED with prednisolone.^[3]^ Long-term use of corticosteroids is not recommended owing to the risk of linear growth impairment and osteoporosis. Deflazacort is a glucocorticoid derived from prednisolone and is reported to be useful in patients with CED, with fewer adverse effects.^[24]^ We applied deflazacort to our patients, and found it to be effective in relieving pain and inflammation, as evidenced by the ESR decrease without any adverse effects. Recently, losartan, an angiotensin II type 1 receptor antagonist that downregulates the expression of TGFβ type 1 and 2 receptors, has been applied to patients with CED.^[10,11]^ It was found to be effective in eliminating incapacitating pain, and in improving muscle strength and lean body mass without obvious adverse effects. However, only 2 patients in our study were able to maintain a good condition with losartan monotherapy. Hypotension with dizziness was observed in 1 patient after the initiation of losartan. Long-term follow-up of losartan therapy in patients with CED is needed to establish its efficacy and safety.

Considering the progressive sclerotic nature of this condition, early identification of each patient is essential. Moreover, corticosteroids or angiotensin II receptor blockers have been described to be effective in the management of CED.^[3]^ Therefore, its early identification with appropriate medical treatment is warranted for the long-term prognosis of each patient. In our study, CED was diagnosed during the pediatric period in only 1 patient, and the disease was diagnosed in the other 3 patients at their second to fourth decade, with severe complications including cranial nerve involvement with visual or otological manifestations, skeletal deformities requiring surgical correction, and osteoporosis. Patient 4 who started treatment since age of 11 years showed a notable improvement in bone scintigraphy which was performed after a year from treatment initiation. Long-term follow-up of this patient will demonstrate the long-term prognosis of early treated patients.

In conclusion, CED must be suspected in patients presenting with pain in the extremities and showing radiologic findings with symmetric cortical thickening of the diaphysis. Bone scintigraphy is a valuable diagnostic tool, and mutation analysis of *TGFB1* is a confirmatory test. Corticosteroids or angiotensin II receptor blockers are applicable for treatment, but there are still some limitations to their safety and efficacy. The treatment approach to modulate TGFβ1 signaling pathway might be an additional way to manage patients with CED.

## Author contributions

**Conceptualization:** B.H. Lee, Y-M. Kim.

**Data curation:** G-H. Kim, Y-M. Kim.

**Formal analysis:** E. Kang, G-H. Kim, Y-M. Kim.

**Investigation:** E. Kang, J-H. Choi.

**Supervision:** B.H. Lee, H-W. Yoo.

**Writing – original draft:** Y-M. Kim.

**Writing – review and editing:** B.H. Lee, H-W. Yoo, J-H. Choi, Y-M. Kim.
